# Molecular structure and phylogenetic analysis of complete chloroplast genomes of medicinal species *Paeonia lactiflora* from Zhejiang Province

**DOI:** 10.1080/23802359.2020.1721372

**Published:** 2020-02-07

**Authors:** Chenshu Gao, Qirui Wang, Zhiqi Ying, Yuqing Ge, Rubin Cheng

**Affiliations:** a College of Pharmaceutical Science, Zhejiang Chinese Medical University, Hangzhou, China;; b The First Affiliated Hospital, Zhejiang Chinese Medical University, Hangzhou, China

**Keywords:** *Paeonia lactiflora*, chloroplast genome, phylogenetic analysis, simple sequence repeats

## Abstract

*Paeonia lactiflora* is a geo-authentic and superior medicinal plant in Zhejiang province. Here, we report the complete chloroplast genome sequence of *P. lactiflora*. The total genome size of *P. lactiflora* is 153,405 bp in length, including a small single-copy (SSC) region of 16,969 bp, a large single-copy (LSC) region of 84,340 bp separated by a pair of inverted repeats (IRs) of 26,048 bp. The overall annotated gene number is 109, containing 76 protein-coding genes, 29 tRNAs and 4 rRNAs. The entire GC content of *P. lactiflora* is 38.43%, with the highest GC content of 42.99% in IR region. A total of 52 simple sequence repeats are identified in the cp genome of *P. lactiflora*. Phylogenetic analysis indicated a sister relationship between *P. lactiflora* and *P. veitchii*, and supported a unique evolutionary status of Family Paeoniaceae. This work provides a valuable genetic resource to develop robust markers and investigate the population genetics diversities for this famous medicinal species.

*Paeonia lactiflora* belonging to the Paeoniaceae family is an important ornamental and medicinal plant (Guo 2002). The root of *P. lactiflora* is a member of geo-authentic crude drugs “Zhebawei” in Zhejiang Province and has been used to treat various inflammatory diseases (He and Dai 2011). In addition, the seeds of *P. lactiflora* could be a good bio-resource for edible oil and total monoterpene glycosides (Liu et al. 2017). However, there have been more than 600 cultivars of *P. lactiflora* species worldwide, whose chemical compositions and pharmacological activities vary widely among different cultivars. It is necessary to establish typical molecular markers of the medical germplasm of *P. lactilflora* of Zhejiang Province for intraspecific identification. Here, we report the complete chloroplast genome of *P. lactiflora* from Zhejiang Province to provide a genomic resource for molecular marker development and clarify the phylogenetic relationship of this plant with other species in Paeoniaceae family.

Total genomic DNA was extracted from fresh leaves of *P. lactiflora* sample of ZJSC JLS2019 collected from Suichang area in Zhejiang Province of China (28°23′22.01″N, 118°51′1.66″E). The specimen was deposited in the collection center of Zhejiang Chinese Medical University. Total genomic DNA was sequenced using the Illumina Hiseq Platform according to our previous studies (Ying et al. 2019). The complete chloroplast genome of *P. lactiflora* was assembled from the extracted cp-like reads by metaSPAdes with the well-defined complete chloroplast genome of *Paeonia rockii* as the reference (Nurk et al. 2017). The assembled chloroplast genome was annotated using the online tool GeSqe and further corrected by BLAST (Tillich et al. 2017). The final complete cp genome of *P. lactiflora* was submitted to GenBank with the accession number of MN868412.

The chloroplast genome of *P. lactiflora* exhibited a typical and conserved quadripartite structure with the length of 153,405 bp, consisting of a pair of IRs (26,048 bp) separated by the LSC (84,340 bp) and SSC regions (16,969 bp). The overall GC content of the cp in *P. lactiflora* was 38.43%, and the corresponding GC contents for IR, LSC and SSC regions are 42.99%, 36.76% and 37.72%, respectively. Total 109 genes were identified in the chloroplast including76 protein-cording genes, 29 tRNA genes and four rRNA genes. The complete cp genome of *P. lactiflora* contains 50.47% protein-coding sequences and includes 19 duplicated genes in IR region. Moreover, a total of 52small single repeats (SSR) are identified in *P. lactiflora*, ranging from 10 bp to 21 bp.

Phylogenetic analysis was performed using the complete cp genomes of *P. lactiflora* with those of 12 species in family Paeoniaceae as well as 18 species in family Ranunculaceae by maximum-likelihood (ML) method of MEGA 7.0 (Kumar et al. 2016). Our results demonstrated a sister relationship between *P. lactiflora* and *P. veitchii*, indicating a close relationship between the two species (Figure 1). Furthermore, the monophyletic group formed by Paeoniaceae species and the monophyletic clade consisted by Ranunculaceae species did not cluster together, but was separated by the outgroup species, providing molecular evidences for the isolation of Paeonia from Ranunculaceae Family. The complete cp-genome information of *P. lactiflora* would contribute the development of molecular markers and understanding of evolutionary history and cultivation strategy in Family Paeoniaceae.

**Figure 1.**
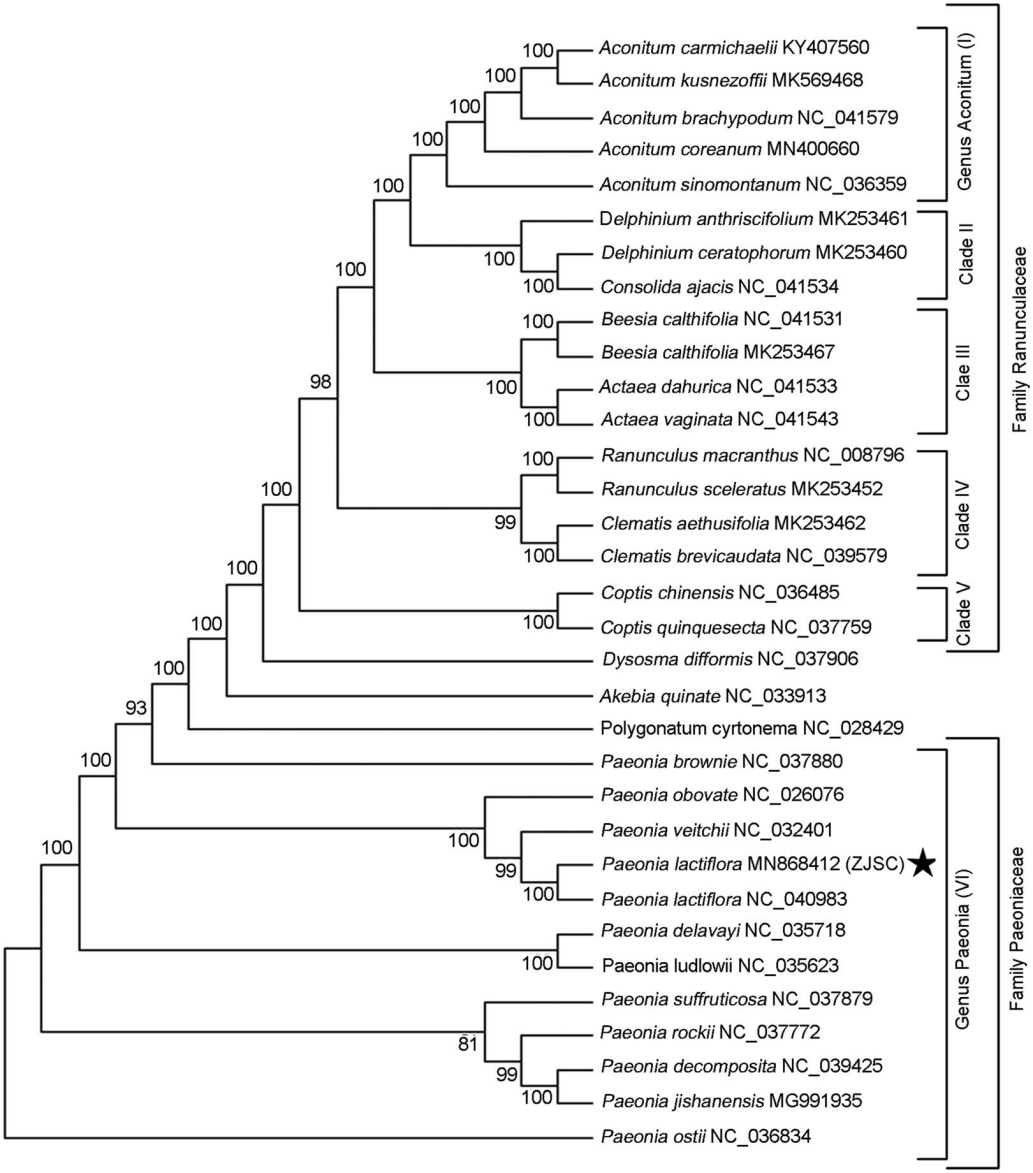
Phylogenetic tree position of the newly sequenced Paeonia lactiflora from Zhejiang Province inferred by ML analysis of other representative species from Paeoniaceae and Ranunulacea based on the complete chloroplast genomes. The species of *Akebia quinate*, *Dysosma difformis* and *Polygonatum cyrtonema* were used as the out-group. Numbers on the nodes are bootstrap values from 100 replicates. The GenBank accession numbers were listed following the species name.
